# An Enhanced Direct Position Determination of Mixed Circular and Non-Circular Sources Using Moving Virtual Interpolation Array

**DOI:** 10.3390/s24206718

**Published:** 2024-10-18

**Authors:** Zhaobo Wang, Jun Zhang, Hui Guo, Yingjie Miao

**Affiliations:** Nanjing Electronic Equipment Institute, Nanjing 210000, China; leviwong737@gmail.com (Z.W.); wangzb3327@163.com (H.G.); wuximyj@gmail.com (Y.M.)

**Keywords:** direct position determination (DPD), sparse array, non-circular and circular signals, virtual array interpolation, low-rank structured covariance reconstruction (LRSCR), unitary transformation method

## Abstract

In this study, a moving single-station direct position determination (DPD) algorithm based on virtual interpolated arrays is proposed. Existing moving single-station algorithms face challenges such as the incomplete utilization of sparse array apertures and insufficient consideration of mixed circular and non-circular signals. To address these issues, we propose an enhanced gridless DPD algorithm, suitable for multiple mixed circular and non-circular sources. Through constructing a non-zero unconjugated covariance matrix from the non-circular components of the mixed signals, the data dimensionality is expanded, and the gridless method is used to fill the voids in the coarray, significantly improving localization performance. Additionally, a unitary transformation method is applied to reduce computational complexity. This method transforms complex operations into real operations by applying unitary transformations to steering vectors and subspaces. Simulation results demonstrate that the proposed algorithm offers significant advantages in terms of array degrees of freedom and localization accuracy.

## 1. Introduction

Passive localization technology refers to techniques that determine the location of an emission source using only electromagnetic information received by an observation platform, without emitting electromagnetic signals [1,2,3]. Traditional passive localization methods typically use a two-step approach to estimate the source location. First, mathematical models are used to estimate localization parameters such as phase difference, time difference of arrival, angle of arrival, and Doppler frequency. These parameters are then associated, and equations are solved to determine the source’s position [4,5,6,7,8,9]. However, these methods suffer from poor robustness in low-signal-to-noise-ratio (SNR) environments and difficulty in parameter pairing in multi-emitter scenarios. To address these issues, direct localization methods, which estimate the source location directly from the received signal [10,11,12,13,14], have been proposed. Compared to multi-station direct localization algorithms [15,16,17], motion-based single-station direct localization algorithms offer the advantage of not requiring time-frequency synchronization between stations or the design of data transmission, thereby reducing system complexity. Additionally, these algorithms allow the motion platform to construct arbitrary observation configurations, greatly enhancing system flexibility. This paper focuses primarily on the research of motion-based single-station direct localization algorithms.

Weiss and Amour first introduced the concept of direct localization algorithms [12], which process multi-observation data models based on the maximum likelihood method. However, since the maximum likelihood method requires a multidimensional search of the nonlinear cost function, it significantly increases the algorithm’s complexity. In 2008, Bruno Demissie and others used the MUSIC algorithm to fuse observation data from multiple time slots to determine the target’s position [18] and provided the Cramér–Rao Lower Bound (CRLB) for direct localization algorithms. In 2016, Weiss and others proposed a direct localization algorithm based on the MVDR method [19]. Unlike subspace-based algorithms, this method does not require the prior estimation of the number of signal sources, thus avoiding performance degradation caused by incorrect source number estimation. However, this method is slightly less accurate than subspace-based algorithms in high-signal-to-noise-ratio (SNR) environments. Due to the presence of a large number of non-circular signals in modern communication systems, non-circular signals have the advantage of a non-zero unconjugated covariance matrix, which not only enhances positioning accuracy but also extends the aperture of the array. In 2017, Yin and others [20] incorporated the non-circular signal model into the direct localization framework, effectively extending the array aperture using non-circular signals. However, this algorithm may fail when circular signals are present in the incident signals. Additionally, several algorithms have been studied for direct localization in different scenarios, such as the study of intermittent signal emission by sources [21] and algorithms utilizing Doppler information for direct localization [22].

However, the methods mentioned above are limited to studies on uniform arrays and offer only limited improvements in an array’s degrees of freedom and observation accuracy. Sparse arrays are artificially designed non-uniform arrays that have the advantages of high direction-finding accuracy and high degrees of freedom. They mainly include coprime arrays [23] and nested arrays [24]. After extensive research, sparse arrays have evolved into various configurations such as generalized coprime arrays [25], super nested arrays [26], and augmented coprime arrays [27], further improving the performance of the original sparse arrays. To further enhance the degrees of freedom and localization accuracy of the single-station observation system, reference [28] integrated a coprime array into the motion platform. This system vectorizes the covariance matrix of the received signals and constructs a virtual difference array, effectively filling the gaps in the original array to some extent, thereby improving the localization accuracy and degrees of freedom. However, these types of algorithms only utilize the largest continuous portion of the coarray, discarding some information, which leads to a reduction in multi-source observation capabilities and localization accuracy. Compared to traditional direct localization algorithms, sparse reconstruction-based algorithms can fully exploit the received information from the array, offering stronger advantages in terms of degrees of freedom, resolution, and localization accuracy [29].

However, sparse algorithms based on $l_{p}$ norm grid-based processing methods [30,31,32,33,34,35], while demonstrating good performance in low-SNR and coherent signal environments [36], suffer from a key limitation: the sparse solution is constrained to the predefined grid, which can lead to mismatch with the true solution and often results in local optimal solutions [37]. In real-world scenarios, the positions of radiation sources are often unknown, and pre-set discrete grids cannot guarantee that the true positions will fall on them. As a result, two types of algorithms have been developed to address grid mismatch: off-grid algorithms [38,39,40] and gridless algorithms [41,42,43,44]. A prominent off-grid algorithm is the Off-Grid Sparse Bayesian Learning (OGSBL) algorithm [45], which performs a first-order Taylor expansion of the array’s true steering vector on the nearby grid. In adding the first-order Taylor expansion term of the true steering vector and an error weighting coefficient to the nominal steering vector corresponding to the predefined grid, the influence of the grid is reduced. While this method improves estimation accuracy, the large number of parameters and complex iterative process increases the computational complexity. Moreover, when quantization errors are large, the first-order approximation alone cannot adequately fit the array steering vector, leading to algorithm failure [46]. For direct localization algorithms, covariance reconstruction is required at each observation position, and the use of off-grid methods significantly increases computational complexity [47]. In recent years, gridless methods based on covariance fitting and atomic norm minimization [41,42,43,44] have effectively addressed the grid mismatch problem. Tang and colleagues [41,42] proposed gridless Direction-of-Arrival (DOA) estimation methods using the atomic norm, including approaches based on Semi-Definite Programming (SDP) and atomic norm soft thresholding. Additionally, MISHRA introduced an atomic norm method incorporating prior knowledge of the signal [48], and Zhou and others [43] proposed using the atomic norm to complete coarrays for DOA estimation. Wu and colleagues [49] further applied low-rank matrix reconstruction techniques to achieve gridless parameter estimation for multiple signals.

This paper, based on the coprime array model, proposes a motion-based single-station direct position determination (DPD) algorithm capable of localizing both circular and non-circular signals. Building on the fundamental idea of covariance data fusion, the algorithm employs a gridless method to fill the gaps in both the sum and difference coarray. Then, based on the reconstructed equivalent array, it separates different signals by leveraging differences in their degree of non-circularity. To the best of our knowledge, most existing algorithms focus primarily on circular signals. This algorithm, however, expands the aperture of sparse arrays utilizing the non-circular components of the received signals. By employing an improved Subspace Data Fusion (SDF) algorithm, it achieves high-precision localization for multiple signal sources. Additionally, the algorithm reduces computational complexity using unitary transformation to shift operations from the complex domain to the real domain.

The contributions of this study are as follows:

(1) In leveraging the characteristic that the unconjugated covariance matrix of the non-circular component of the signal is non-zero, the sum coarray is constructed. This enhances the array’s degrees of freedom and localization accuracy compared to single-station DPD algorithms that integrate only the difference coarray.

(2) The virtual interpolated array technique is employed to fully utilize all actual observations, filling the discontinuities in the virtual sum and difference coarray, thus increasing the information utilization efficiency of the virtual array.

(3) Based on the recovered virtual interpolated array, the direct localization cost function for mixed signals is derived. The cost function is further improved using a unitary transformation, converting operations from the complex domain to the real domain, which effectively reduces the computational complexity of the algorithm.

The remaining chapters of this paper are organized as follows: Section 2 introduces the DPD localization model. Section 3 first integrates the sparse array into the DPD motion platform and then constructs the models for the sum coarray and difference coarray of the sparse array. Subsequently, a gridless method is used to fill the gaps in the virtual array, and the cost function for mixed circular and non-circular signals is derived. Section 4 presents the results of numerical simulations. Section 5 further discusses the significance and potential of the algorithm through simulation analysis. Finally, Section 6 provides a summary of the paper.

Notation: T denotes the transpose; ∗ denotes the conjugate; H denotes the conjugate transpose; E(·) represents the expectation; ⊙ denotes the Khatri–Rao product; ⊗ denotes the Kronecker product; ∘ denotes the Hadamard product; |S| represents the cardinality of set *S*; ${\left[\cdot \right]}_{i}$ denotes the *i*-th element of a vector; ${∥\cdot ∥}_{F}$ represents the Frobenius norm; vec denotes the vectorization operation, which arranges a matrix into a column vector by stacking its columns; Tr(·) denotes the trace of a matrix; and I represents the identity matrix.

## 2. DPD Model with a Moving Array

### DPD Model Based on Coprime Array

**Figure 1:**
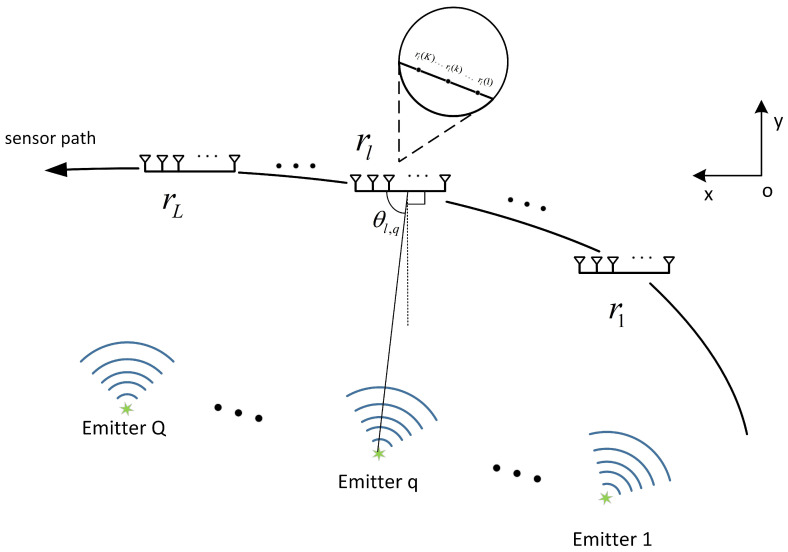
Geometry of a single moving platform and emitters.

Consider the scenario shown in Figure 1: a moving sensor platform receives spatially separable narrowband signals emitted by *Q* stationary sources, where the position of each source is denoted as $p_{q}={\left(x_{q},y_{q}\right)}^{T},q=1,\ldots ,Q$. These sources transmit narrowband signals with a central wavelength of λ, and the signals are mutually independent. The sensor platform is equipped with *M* omnidirectional antennas arranged in a coprime array configuration. During its movement, the platform collects data at *L* different positions with *K* snapshots at each position. The position of the platform at each sampling point is denoted as $p_{l}={\left(x_{l},y_{l}\right)}^{T},l=1,\ldots ,L$. In assuming that the platform moves slowly enough such that Doppler shifts can be ignored and the channel parameters remain constant throughout the process, the DOA information of the sources at each position can be considered approximately unchanged. Therefore, the signal received at the *l*-th position during the *k*-th snapshot, denoted as $r_{l}\left(k\right)\in C^{M\times 1}$, is expressed as

(1) $$r_{l}\left(k\right)=\sum_{q=1}^{Q}a_{l}\left(p_{q}\right)s_{l,q}\left(k\right)+n_{l}\left(k\right)\;\;\;k=1,2,\ldots ,K$$

where $a_{l}\left(p_{q}\right)$ represents the antenna steering vector for the *q*-th source when the platform moves to the *l*-th position, and $s_{l,q}\left(k\right)$ denotes the *k*-th transmitted signal of the *q*-th source sampled when the platform is at the *l*-th position. $n_{l}\left(k\right)\in C^{M\times 1}∼N\left(0,\delta_{n}^{2}I_{M}\right)$ represents the noise signal received at the *l*-th position by the platform, where the noise is assumed to be independent of the transmitted signals from the sources. Reorganizing the above equations into matrix form, we have

(2) $$r_{l}\left(k\right)=A_{l}\left(p\right)s_{l}\left(k\right)+n_{l}\left(k\right)\in C^{M\times 1}$$

The array manifold is defined as

(3) $$A_{l}\left(p\right)=\left[a_{l}\left(p_{1}\right),a_{l}\left(p_{2}\right),\ldots ,a_{l}\left(p_{Q}\right)\right]\in C^{M\times Q}$$

where

(4) $$a_{l}\left(p_{q}\right)={\left[e^{-j{2\pi v}_{l}^{T}\left(p_{q}\right)d_{1}},e^{-j{2\pi v}_{l}^{T}\left(p_{q}\right)d_{2}},\ldots ,e^{-j{2\pi v}_{l}^{T}\left(p_{q}\right)d_{M}}\right]}^{T}$$

where $d_{m}$ represents the position vector of the *m*-th sensor relative to the reference sensor, and $\Delta r_{l}\left(p_{q}\right)=p_{l}-p_{q}={\left(\Delta x_{l}\left(p_{q}\right),\Delta y_{l}\left(p_{q}\right)\right)}^{T}$ represents the position vector from the radiation source to the antenna. Thus, the wavenumber vector is given by

(5) $$v_{l}^{T}\left(p_{q}\right)=\frac{1}{\lambda }\frac{\Delta r_{l}\left(p_{q}\right)}{∥\Delta r_{l}\left(p_{q}\right){∥}_{2}}$$

Through combining the data from *L* time periods, the received signal is obtained as follows:

(6) $$r\left(k\right)={\left[\begin{array}{c} r_{1}{\left(k\right)}^{T},r_{2}{\left(k\right)}^{T},\ldots ,r_{L}{\left(k\right)}^{T} \end{array}\right]}^{T}\in C^{ML\times 1}$$

The transmitted signal is given by

(7) $$s\left(k\right)={\left[\begin{array}{c} s_{1}{\left(k\right)}^{T},s_{2}{\left(k\right)}^{T},\ldots ,s_{L}{\left(k\right)}^{T} \end{array}\right]}^{T}\in C^{LQ\times 1}$$

The array manifold is given by

(8) $$A=\left[\begin{array}{ccc} A_{1}\left(p\right) & \cdots & 0\\ \vdots & \ddots & \vdots \\ 0 & \cdots & A_{L}\left(p\right) \end{array}\right]\in C^{LM\times LQ}$$

Thus, we have

(9) r(k)=As(k)+n(k)

where $n\left(k\right)∼N\left(0,\delta_{n}^{2}I_{LM}\right)$.

## 3. Proposed Algorithm

**Figure 2:**
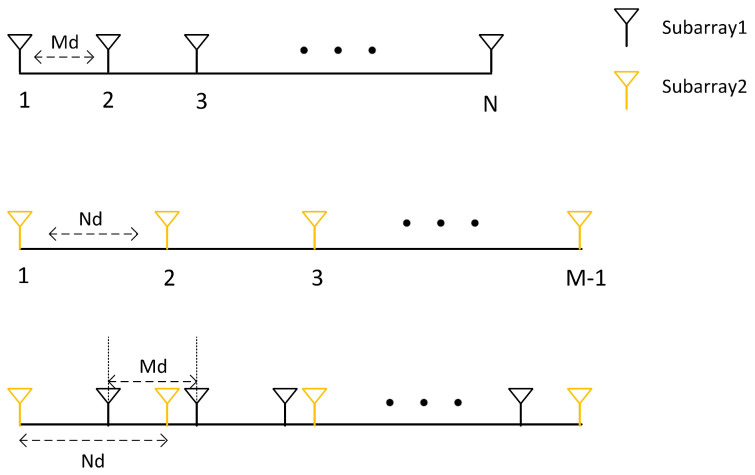
Coprime array configuration.

The platform integrates a coprime sensor array consisting of two subarrays, as shown in Figure 2. Subarray one consists of *N* sensors with a spacing of Md, and subarray two consists of *M* sensors with a spacing of Nd, where *M* and *N* are coprime, and $d=\frac{\lambda }{2}$. The subarrays are arranged in a straight line and share the same reference element, so the array has a total of M+N−1 sensors. The positions of the sensors can be expressed as

(10) $$\begin{array}{cc} L_{\mathrm{CA}} & =L_{\mathrm{CA}}^{\left(1\right)}\cup L_{\mathrm{CA}}^{\left(2\right)}\\ \; & =\{nMd,n\in (0,N-1)\}\cup \{mNd,m\in (0,M-1)\} \end{array}$$

Circularity and non-circularity [50] are important properties of random signals. Modern communication systems use a large number of non-circular signals, such as BPSK, UQPSK, MSK, and other modulated signals. Since the unconjugated covariance matrix of non-circular signals is non-zero, it is possible to enhance the degrees of freedom of the array by extending the conjugate of the received signal. It is important to note that in array signal processing, the signal does not need to meet the condition of non-circularity strictly; satisfying the condition of pseudo-non-circularity is sufficient. The condition for pseudo-circularity is shown as follows [51]:

(11) $$E\left[se^{j\phi }\right]=E\left[s\right]$$

(12) $$E\left[se^{j\phi }{\left(se^{j\phi }\right)}^{*}\right]=E\left[ss^{*}\right]$$

(13) $$E\left[se^{j\phi }se^{j\phi }\right]=E\left[ss\right]$$

As seen from Equation (13), the unconjugated covariance of non-circular signals is non-zero. Most existing localization algorithms utilize the covariance information of the signal while ignoring the unconjugated covariance information, which leaves room for improvement in the array’s degrees of freedom and accuracy. For non-circular signals, the following condition is satisfied [51]:

(14) $$E\left[s\left(k\right)s\left(k\right)\right]=\rho e^{j\phi }E\left[s\left(k\right)s^{*}\left(k\right)\right]$$

where ϕ represents the non-circular phase, and ρ denotes the non-circularity rate, with a value in the range 0<ρ≤1. For Maximal Non-circularity Rated Signals, ρ=1, and for Common Non-circularity Rated Signals, 0<ρ<1.

For *Q* uncorrelated sources, the unconjugated covariance matrix of the signal is given by

(15) $$\begin{array}{cc} R_{S}^{\prime } & =E\left[s\left(k\right)s^{T}\left(k\right)\right]\\ \; & =\mathrm{diag}\left\{E\left[s_{1}\left(k\right)s_{1}\left(k\right)\right],E\left[s_{2}\left(k\right)s_{2}\left(k\right)\right],\ldots ,E\left[s_{Q}\left(k\right)s_{Q}\left(k\right)\right]\right\}\\ \; & =\mathrm{diag}\left\{\rho_{1}e^{j\phi_{1}}E\left[s_{1}\left(k\right)s_{1}^{*}\left(k\right)\right],\rho_{2}e^{j\phi_{2}}E\left[s_{2}\left(k\right)s_{2}^{*}\left(k\right)\right],\ldots ,\rho_{q}e^{j\phi_{q}}E\left[s_{Q}\left(k\right)s_{Q}^{*}\left(k\right)\right]\right\}\\ \; & ≜{\mathrm{PBR}}_{S} \end{array}$$

where $P=\mathrm{diag}\{\rho_{1},\rho_{2},\ldots ,\rho_{Q}\}$ is a diagonal matrix composed of the non-circularity rates of each signal. For Maximal Non-circularity Rated Signals, $P=I_{Q}$, while $B\;=\;\mathrm{diag}\{e^{j\phi_{1}},e^{j\phi_{2}},\ldots ,e^{j\phi_{Q}}\}$ is a diagonal matrix composed of the non-circular phases of each signal.

Based on (2) and (15), the expression for the unconjugated covariance matrix of the received signal can be derived as

(16) $$R^{\prime }=E\left[r(k)r(k)\right]=AR_{S}A^{T}+E\left[n\left(k\right)n^{T}\left(k\right)\right]=A{\mathrm{PBR}}_{S}A^{T}.$$

In practical applications, the covariance and unconjugated covariance matrix of each batch of received signals are replaced by the sample covariance, which is given by

(17) $${\hat{R}}_{l}=\frac{1}{K}\sum_{k=1}^{K}r_{l}\left(k\right)r_{l}^{H}\left(k\right),\;{\hat{R}}_{l}^{\prime }=\frac{1}{K}\sum_{k=1}^{K}r_{l}\left(k\right)r_{l}^{T}\left(k\right).$$

By performing conjugate augmentation on the received signal vector, we obtain

(18) $$\tilde{r}\left(k\right)={\left[\begin{array}{c} r^{T}\left(k\right),r^{H}\left(k\right) \end{array}\right]}^{T}$$

The covariance matrix of the extended signal vector ${\tilde{r}}_{l}\left(k\right)$ for the *l*-th batch is

(19) $${\tilde{R}}_{l}=E\left[{\tilde{r}}_{l}\left(k\right){\tilde{r}}_{l}^{H}\left(k\right)\right]=\left[\begin{array}{cc} R_{l} & R_{l}^{\prime }\\ R_{l}^{\prime *} & R_{l}^{*} \end{array}\right]$$

Vectorizing $R_{l}$ in Equation (19), we obtain

(20) $$\begin{array}{cc} z_{d,l} & =\mathrm{vec}(R_{l})\\ \; & =\left(A_{l}^{*}⊙A_{l}\right)\rho +\sigma_{n}^{2}I_{e}\\ \; & =B_{l}\rho +\sigma_{n}^{2}I_{e} \end{array}$$

where ⊙ denotes the Khatri–Rao product, $I_{e}={\left[e_{1}^{T},e_{2}^{T},...,e_{M+N-1}^{T},e_{1}^{T},e_{2}^{T},...,e_{M+N-1}^{T}\right]}^{T}$, where $e_{1}^{T}$ represents a row vector with the first element as 1 and the remaining elements as 0. $\rho ={\left[\sigma_{1}^{2},\sigma_{2}^{2},\ldots ,\sigma_{Q}^{2}\right]}^{T}$, and $b_{l}\left(p_{q}\right)=a_{l}^{*}\left(p_{q}\right)⊗a_{l}\left(p_{q}\right)$. The matrix $A_{l}$ is given by $A_{l}=\left[b_{l}\left(p_{1}\right),b_{l}\left(p_{2}\right),\ldots ,b_{l}\left(p_{Q}\right)\right]$.

Vectorizing $R_{l}^{\prime }$ in Equation (19), we obtain

(21) $$\begin{array}{cc} z_{s,l} & =\mathrm{vec}(R_{l}^{\prime })\\ \; & =\left(A_{l}⊙A_{l}\right)\rho^{\prime }\\ \; & =B_{l}^{\prime }\rho \end{array}$$

where $\rho^{\prime }={\left[\rho_{1}e^{j\phi_{1}}\sigma_{1}^{2},\rho_{2}e^{j\phi_{2}}\sigma_{2}^{2},...,\rho_{Q}e^{j\phi_{Q}}\sigma_{Q}^{2}\right]}^{T}$.

Thus, from Equations (20) and (21), the following virtual difference coarray and sum coarray can be derived: $D_{diff}=\left\{u_{m}-u_{n}|m,n=0,1,\ldots ,M+N-1\right\}$, $D_{sum}^{+}=\left\{u_{m}+u_{n}|m,n=0,1,\ldots ,M+N-1\right\}$.

**Figure 3:**
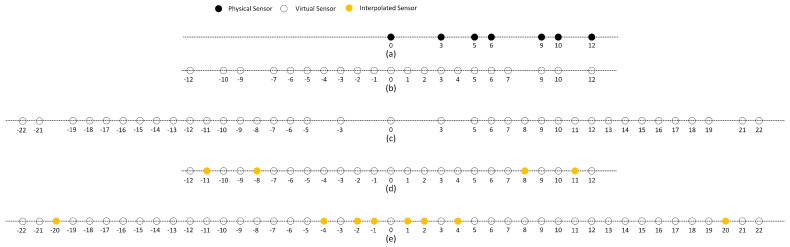
Schematic example of array structure with M = 3 and N = 5. (**a**) Physical coprime array. (**b**) The DCA of coprime array $D_{diff}$. (**c**) The SCA of coprime array $D_{sum}$. (**d**) The interpolated array of (**b**) $D_{I}$. (**e**) The interpolated array of (**c**), $S_{I}$.

However, the virtual array constructed in this way will have gaps. Traditional methods can only utilize the largest continuous portion of the virtual array, discarding the non-continuous parts, which leads to information loss. If we fill in the gaps of the non-continuous elements in the array, we can maximize the utilization of all the information from the elements. Let

(22) $${\left[z_{I}\right]}_{i}=\left\{\begin{array}{cc} {\left[z_{v}\right]}_{i} & i\in D_{diff}\\ 0 & i\in D_{I}-D_{diff} \end{array}\right.$$

(23) $${\left[s_{I}\right]}_{i}=\left\{\begin{array}{cc} {\left[s_{v}\right]}_{i} & i\in D_{sum}^{+}\\ 0 & i\in S_{I}-D_{sum}^{+} \end{array}\right.$$

where ${\left[z_{I}\right]}_{i}$ and ${\left[s_{I}\right]}_{i}$ represent the virtual array received signals of $D_{I}$ and $S_{I}$, while ${\left[z_{v}\right]}_{i}$ and ${\left[s_{v}\right]}_{i}$ represent the virtual array received signals of $D_{diff}$ and $D_{sum}^{+}$. This approach maximizes the utilization of the virtual elements of the sum and difference coarray. The corresponding interpolation process is illustrated in Figure 3.

Note: The generally defined sum coarray, $D_{sum}$, is $D_{sum}^{+}\cup D_{sum}^{-}$, but $D_{sum}^{-}$ corresponds to $R_{l}^{\prime *}$, and the information it contains is identical to that of $D_{sum}^{+}$, which corresponds to $R_{l}^{\prime }$. Therefore, unless otherwise specified, the sum coarray in this paper refers to $D_{sum}^{+}$.

### 3.1. Gridless Recovery Based on Array Interpolation

Although the SCA (sum coarray array) and DCA (difference coarray array) contain gaps, these gaps can be filled using array interpolation methods. The corresponding schematic diagram is shown in Figure 3. The work by [43] uses subarray division techniques to establish the relationship between the virtual coarray received signals of the difference coarray and the covariance matrix of the equivalent array’s received signals. Since the covariance matrix of the ideal signal satisfies a Toeplitz structure, we use Equation (22) to construct the covariance matrix of the equivalent array directly:

(24) $$R_{D}=\begin{array}{c} \left[\begin{array}{cccc} {\left[z_{I}\right]}_{J} & {\left[z_{I}\right]}_{J+1}^{*} & \ldots & {\left[z_{I}\right]}_{2J-1}^{*}\\ {\left[z_{I}\right]}_{J+1} & {\left[z_{I}\right]}_{J} & \ldots & {\left[z_{I}\right]}_{2J-2}^{*}\\ \vdots & \vdots & \ddots & \vdots \\ {\left[z_{I}\right]}_{2J-1} & {\left[z_{I}\right]}_{2J-2} & \ldots & {\left[z_{I}\right]}_{J} \end{array}\right] \end{array}$$

where $J=(\left|D_{I}\right|+1)/2$. We can thus formulate the following atomic norm minimization (ANM) problem:

(25) $$\begin{array}{cc} \min_{u\in D^{J}} & {∥u∥}_{A}\\ s.t. & \left\{\begin{array}{c} ∥T\left(u\right)\circ G-R_{D}{∥}_{F}^{2}\leq \epsilon ,\\ T\left(u\right)\geq 0, \end{array}\right. \end{array}$$

where $T\left(u\right)=\sum_{q=1}^{Q}b_{I}\left(p_{q}\right)*b_{I}{\left(p_{q}\right)}^{H}*\rho_{q}\;$ represents a Hermitian positive semi-definite matrix generated from the first column u of $R_{D}$. Here, $b_{I}\left(p_{q}\right)=\left[e^{-j2\pi v^{T}\left(p_{q}\right)d_{J}},\ldots ,\right.{\left.e^{-j2\pi v^{T}\left(p_{q}\right)d_{2J-1}}\right]}^{T}$ represents the steering vector of the equivalent array. G is a selection matrix that ensures $T\left(u\right)$ matches the zero elements of $R_{D}$. Since $R_{D}$ contains all the collected information, $T\left(u\right)$ does not extend the available information.

Due to the difficulty in solving Equation (25), it is convexly relaxed to the $l_{1}$ atomic norm.

(26) $$\begin{array}{cc} \min_{x>0,u\in D^{J}} & \frac{1}{2}{∥T\left(u\right)\circ G-R_{D}∥}_{F}^{2}+\mu \mathrm{Tr}\left(T\left(u\right)\right)\\ s.t. & \left[\begin{array}{cc} x & {\left(z_{I}\right)}^{H}\\ z_{I} & T(u) \end{array}\right]⪰0 \end{array}$$

where

(27) $$z_{I}=\sum_{q=1}^{Q}\rho_{q}*b_{I}\left(p_{q}\right)$$

Similar to Equation (24), we can construct the relationship between the virtual elements of the sum coarray’s received signal and the unconjugated covariance of the equivalent array’s received signal. Since the unconjugated covariance matrix of the ideal signal satisfies a Hankel structure, we use Equation (23) to construct the unconjugated covariance matrix of the equivalent array directly:

(28) $$R_{S}=\begin{array}{c} \left[\begin{array}{cccc} {\left[s_{I}\right]}_{1} & {\left[s_{I}\right]}_{2} & \ldots & {\left[s_{I}\right]}_{J}\\ {\left[s_{I}\right]}_{2} & {\left[s_{I}\right]}_{3} & \ldots & {\left[s_{I}\right]}_{J+1}\\ \vdots & \vdots & \ddots & \vdots \\ {\left[s_{I}\right]}_{J} & {\left[s_{I}\right]}_{J+1} & \ldots & {\left[s_{I}\right]}_{2J-1} \end{array}\right] \end{array}$$

where $J=(\left|S_{I}\right|+1)/2$. It is worth mentioning that after array interpolation, the number of equivalent elements in both the sum coarray and the difference coarray becomes the same, i.e., $\left|S_{I}\right|=\left|D_{I}\right|$.

For the recovery of the unconjugated covariance, we employ low-rank structured covariance reconstruction (LRSCR), specifically

(29) $$\begin{array}{cc} \min_{s\in S^{J}} & \mathrm{rank}(H(s))\\ s.t. & {∥H\left(s\right)\circ G^{\prime }-R_{S}∥}_{F}^{2}\leq \epsilon , \end{array}$$

where $H\left(s\right)=\sum_{q=1}^{Q}b_{I}\left(p_{q}\right)*b_{I}{\left(p_{q}\right)}^{T}*\rho_{q}^{\prime }\;$ represents a Hankel matrix generated from the elements s of $S_{I}$. Equation (29) can be convexly relaxed into the following nuclear norm minimization problem:

(30) $$\min_{s\in S^{J}}\frac{1}{2}{∥H\left(s\right)\circ G^{\prime }-R_{S}∥}_{F}^{2}+\mu {∥H(s)∥}_{*}$$

### 3.2. Positioning Estimation for Circular and Non-Circular Signals

When using Equations (26) and (30) to recover the covariance matrix T(u) and the unconjugated covariance matrix H(s) of the equivalent array, we obtain

(31) $$\begin{array}{cc} {\tilde{R}}_{I} & =\left[\begin{array}{cc} T(u) & H(s)\\ H{\left(s\right)}^{*} & T{\left(u\right)}^{*} \end{array}\right]=\left[\begin{array}{cc} B_{I}R_{s}B_{I}^{H} & B_{I}R_{s}^{\prime }B_{I}^{T}\\ {\left(B_{I}R_{s}^{\prime }B_{I}^{T}\right)}^{*} & {\left(B_{I}R_{s}B_{I}^{H}\right)}^{*} \end{array}\right]\\ \; & =\left[\begin{array}{cc} B_{I}R_{s}B_{I}^{H} & B_{I}PBR_{s}B_{I}^{T}\\ {\left(B_{I}PBR_{s}B_{I}^{T}\right)}^{*} & {\left(B_{I}R_{s}B_{I}^{H}\right)}^{*} \end{array}\right] \end{array}$$

Equation (31) can be rewritten as

(32) $${\tilde{R}}_{I}=\left[\begin{array}{cc} B_{I} & -B_{I}\\ B_{I}^{*}B^{*} & B_{I}^{*}B^{*} \end{array}\right]\left[\begin{array}{cc} \frac{I_{J}+P}{2}R_{S} & 0\\ 0 & \frac{I_{J}-P}{2}R_{S} \end{array}\right]{\left[\begin{array}{cc} B_{I} & -B_{I}\\ B_{I}^{*}B^{*} & B_{I}^{*}B^{*} \end{array}\right]}^{H}$$

For Maximal Non-circularity Rated Signals, Equation (32) can be written as

(33) $${\tilde{R}}_{I}=\left[\begin{array}{c} B_{I}\\ B_{I}^{*}B^{*} \end{array}\right]R_{S}{\left[\begin{array}{c} B_{I}\\ B_{I}^{*}B^{*} \end{array}\right]}^{H}=B_{NC}R_{S}B_{NC}^{H}$$

A typical covariance-based fusion includes the MUSIC, Capon, and maximum likelihood methods. The latter two require a non-circular phase search [52], which significantly increases computational complexity. However, by applying some matrix operations, the MUSIC algorithm can avoid the non-circular phase search [50]. Therefore, this paper adopts the SDF algorithm [18], which is based on MUSIC. As can be seen from Equation (33), the MUSIC method can be used to extract the signal’s DOA information, and its eigenvalue decomposition is performed as follows:

(34) $${\tilde{R}}_{I}=U_{S}\Sigma_{S}U_{S}^{H}+U_{N}\Sigma_{N}U_{N}^{H}$$

According to [50], the spectral function of the MUSIC algorithm for Maximal Non-circularity Rated Signals can be expressed as

(35) $$f_{NC}\left(\theta_{q}\right)=b_{I}^{H}\left(\theta_{q}\right)U_{N1}U_{N1}^{H}b_{I}\left(\theta_{q}\right)-∥b_{I}^{T}\left(\theta_{q}\right)U_{N2}U_{N1}^{H}b_{I}\left(\theta_{q}\right)∥$$

where $U_{N}=\left[\begin{array}{c} U_{N1}\\ U_{N2} \end{array}\right]$.

For circular signals and Common Non-circularity Rated Signals, assuming there are *w* Maximal Non-circularity Rated Signals and *z* mixed Common Non-circularity Rated and circular signals, we can express $R_{s}$ as

(36) $$R_{S}=\left[\begin{array}{cc} R_{S_{w}} & 0\\ 0 & R_{S_{z}} \end{array}\right].$$

Since the non-circularity rate of Maximal Non-circularity Rated Signals is 1, P and B can be written as

(37) P=PwPzPwPz=IwPzIwPz

(38) B=BwBzBwBz

After some matrix transformations, Equation (32) can be rewritten as

(39) $${\tilde{R}}_{I}=B_{wz}\left[\begin{array}{ccc} R_{S_{w}} & 0 & 0\\ 0 & \frac{I_{z}+B_{z}}{2}R_{S_{z}} & 0\\ 0 & 0 & \frac{I_{z}-B_{z}}{2}R_{S_{z}} \end{array}\right]B_{wz}^{H}≜B_{wz}R_{S_{wz}}B_{wz}^{H}$$

where

(40) $$B_{wz}=\left[\begin{array}{ccc} B_{I_{w}} & B_{I_{z}} & B_{I_{z}}\\ B_{I_{w}}^{*}B_{w}^{*} & B_{I_{z}}^{*}B_{z}^{*} & -B_{I_{z}}^{*}B_{z}^{*} \end{array}\right]$$

Based on the form of Equation (39), we can use the MUSIC algorithm to extract the DOA information for mixed Common Non-circularity Rated and circular signals. The DOA estimation spectral function for mixed signals is provided in [53].

(41) $$f_{wz}\left(\theta_{q}\right)=b_{I}^{H}\left(\theta_{q}\right)U_{N1}U_{N1}^{H}b_{I}\left(\theta_{q}\right)$$

It is proven in [50] that

(42) $$U_{N2}=U_{N1}^{*}\Delta$$

Thus, Equation (35) can be written as

(43) $$\begin{array}{cc} f_{NC}\left(\theta_{q}\right) & =b_{I}^{H}\left(\theta_{q}\right)U_{N1}U_{N1}^{H}b_{I}\left(\theta_{q}\right)-∥b_{I}^{T}\left(\theta_{q}\right)U_{N2}U_{N1}^{H}b_{I}\left(\theta_{q}\right)∥\\ \; & =b_{I}^{H}\left(\theta_{q}\right)U_{N1}U_{N1}^{H}b_{I}\left(\theta_{q}\right)-∥b_{I}^{T}\left(\theta_{q}\right)U_{N1}^{*}\Delta U_{N1}^{H}b_{I}\left(\theta_{q}\right)∥ \end{array}$$

From Equation (41), we know that $b_{I}$ is orthogonal to $U_{N1}$, so signals satisfying Equation (41) also satisfy Equation (35).

It is worth mentioning that when using Equation (35) for estimation in scenarios with a small number of snapshots and a low signal-to-noise ratio, spurious peaks may appear at the locations of circular and Common Non-circularity Rated Signals. However, this is beyond the scope of this paper; see [53] for details.

Since non-circular signals significantly extend the array’s degrees of freedom, using Equation (35) for estimation increases computational complexity. Here, we employ a unitary transformation to convert the computation from the complex domain to the real domain, thereby reducing the computational complexity.

When the matrix order is 2d and 2d+1,d=0,1,…, the unitary matrix Q is defined as

(44) $$Q_{2d}=\frac{1}{\sqrt{2}}\left[\begin{array}{cc} I_{d} & jI_{d}\\ \Pi_{d} & -j\Pi_{d} \end{array}\right]$$

(45) $$Q_{2d+1}=\frac{1}{\sqrt{2}}\left[\begin{array}{ccc} I_{d} & 0 & jI_{d}\\ 0^{T} & \sqrt{2} & 0^{T}\\ \Pi_{d} & 0 & -j\Pi_{d} \end{array}\right]$$

where

(46) $$\Pi_{d}=\begin{array}{c} \left[\begin{array}{cccc} 0 & 0 & \ldots & 1\\ 0 & 0 & \ldots & 0\\ \vdots & \vdots & \ddots & \vdots \\ 1 & 0 & \ldots & 0 \end{array}\right] \end{array}\in C^{d\times d}$$

is a counter-diagonal identity matrix.

By left-multiplying $b_{I}$ by the unitary matrix Q, left-multiplying the noise subspace $U_{N1}$ by the unitary matrix Q, and left-multiplying $U_{N2}$ by $Q^{*}$, Equation (35) becomes

(47) $$\begin{array}{cc} f_{NC}\left(\theta_{q}\right) & =b_{I}^{H}\left(\theta_{q}\right)U_{N1}U_{N1}^{H}b_{I}\left(\theta_{q}\right)-∥b_{I}^{T}\left(\theta_{q}\right)U_{N2}U_{N1}^{H}b_{I}\left(\theta_{q}\right)∥\\ \; & =b_{I}^{H}\left(\theta_{q}\right)Q^{H}QU_{N1}U_{N1}^{H}Q^{H}Qb_{I}\left(\theta_{q}\right)-∥b_{I}^{T}\left(\theta_{q}\right)Q^{H}QU_{N2}U_{N1}^{H}Q^{H}Qb_{I}\left(\theta_{q}\right)∥\\ \; & =b_{I,Q}^{H}\left(\theta_{q}\right)U_{N1,Q}U_{N1,Q}^{H}b_{I,Q}\left(\theta_{q}\right)-∥b_{I,Q}^{T}\left(\theta_{q}\right)U_{N2,Q}U_{N1,Q}^{H}b_{I,Q}\left(\theta_{q}\right)∥ \end{array}$$

According to [54], $b_{I,Q}$ is a real vector, thus completing the transformation from the complex domain to the real domain.

Through fusing the data from *L* time periods, a MUSIC-based direct localization spectral function can be constructed as follows:

(48) $$f_{\mathrm{SDF}}\left(p_{q}\right)=\sum_{l=1}^{L}\frac{1}{b_{l,I,Q}^{H}\left(p_{q}\right)U_{N1,Q}U_{N1,Q}^{H}b_{l,I,Q}\left(p_{q}\right)-∥b_{l,I,Q}^{T}\left(p_{q}\right)U_{N2,Q}U_{N1,Q}^{H}b_{l,I,Q}\left(p_{q}\right)∥}$$

The reduction in computational complexity due to the unitary transformation is reflected in the grid search process. Let the number of grid points be $N^{p}$. Without using the unitary transformation, each grid point requires 4L((2J−Q)·J) complex multiplications, resulting in a total of $4N^{p}L\left(\left(2J-Q\right)\cdot J\right)$ complex multiplications for the two-dimensional search. Here, *L* is the number of observation batches, *J* is the number of usable array elements in the equivalent interpolated array, and *Q* is the number of signal sources.

When the Unitary transformation is applied, each grid point requires 8L(2J−Q)·J real multiplications, resulting in a total of $8N^{p}L\left(\left(2J-Q\right)\cdot J\right)$ real multiplications for the two-dimensional search. According to the complexity formulas, it can be seen that the unitary transformation proportionally reduces the complexity of the grid search.

Algorithm 1 illustrates the steps of this algorithm.
**Algorithm 1:** An Enhanced Direct Position Determination of Mixed Circular and Non-Circular Sources Using Moving Virtual Interpolation Array
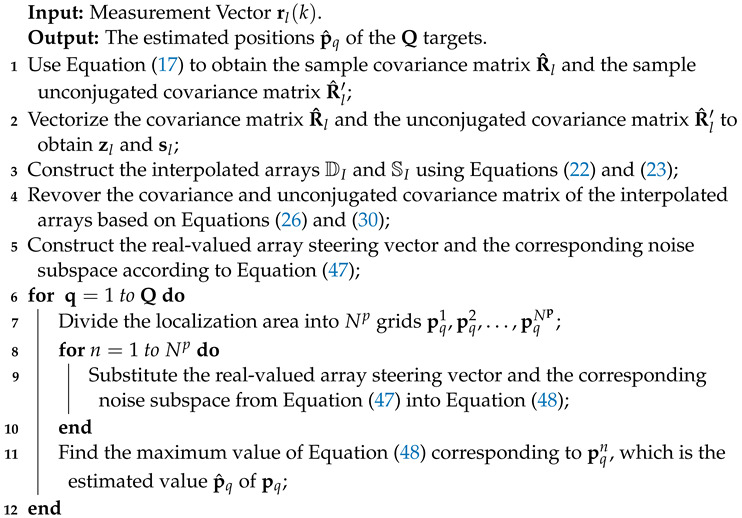


## 4. Simulation Results

In this study, several numerical experiments were conducted to illustrate the effectiveness of the algorithm in localization. All experiments in this study used a mobile platform equipped with a coprime sensor array consisting of M=3 and N=5, for a total of M+N−1=7 sensors, with sensor positions fixed at 0,3d,5d,6d,9d,10d,12d. Without loss of generality, the signal carrier frequency was set to f=3GHz, and the sensor spacing d=0.5λ. The proposed algorithm was compared with the uniform linear array (ULA-SDF) algorithm, the smoothed sum and difference coarray algorithm (SDCA-SDF), the SSR algorithm from [55], and the nuclear norm minimization algorithm proposed in [56]. The regularization parameters in the above algorithms were all set to 0.25, the grid spacing in the SSR algorithm was set to 1°, and the convex optimization problems were solved using the CVX solver in MATLAB R2023a.

### 4.1. Resolution

**Figure 4:**
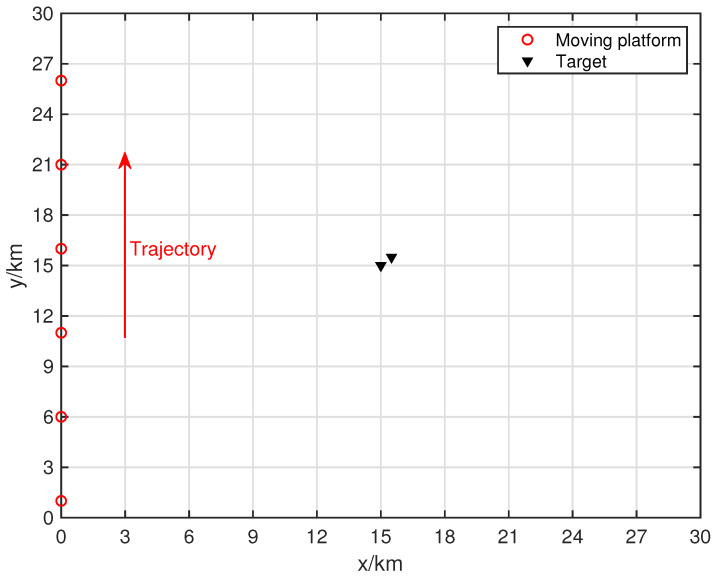
Moving platform scene related to two closed targets.

Figure 4 shows the resolution of each algorithm when dealing with closely spaced targets. The red portions in the figure indicate the platform’s movement trajectory. The target positions were set at (15.0, 15.0) km and (15.5, 15.5) km. The black triangles represent the target locations. In the scenario depicted in Figure 4, the number of snapshots is K=10, and the search area was set to (0 km, 30 km) × (0 km, 30 km), with a grid search density of 500 × 500.

**Figure 5:**
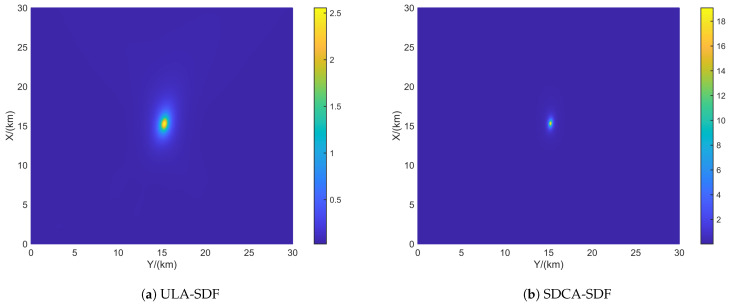

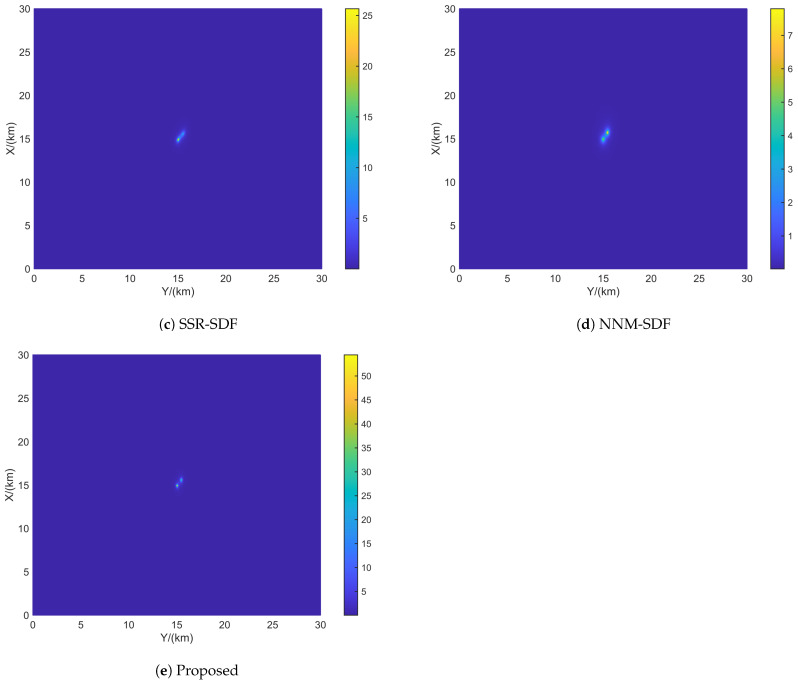
Resolution comparison in terms of the DPD spatial spectrum with the batch number L = 6 and SNR = 10 dB. (**a**) ULA-SDF algorithm. (**b**) SDCA-SDF algorithm. (**c**) SSR-SDF algorithm. (**d**) NNM-SDF algorithm. (**e**) Proposed algorithm.

From Figure 5, it can be observed that ULA- and SDCA-based algorithms show poorer resolution. However, the sparse recovery-based algorithms successfully identify the closely spaced sources set in Figure 4. Among these, the SSR-based algorithm and the algorithm proposed in this paper demonstrate the best performance in identifying the targets.

**Figure 6:**
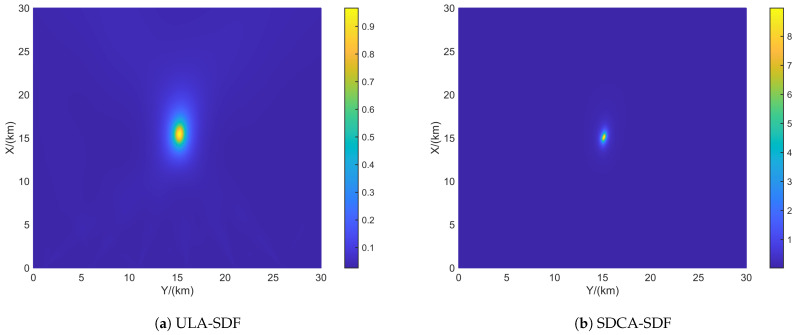

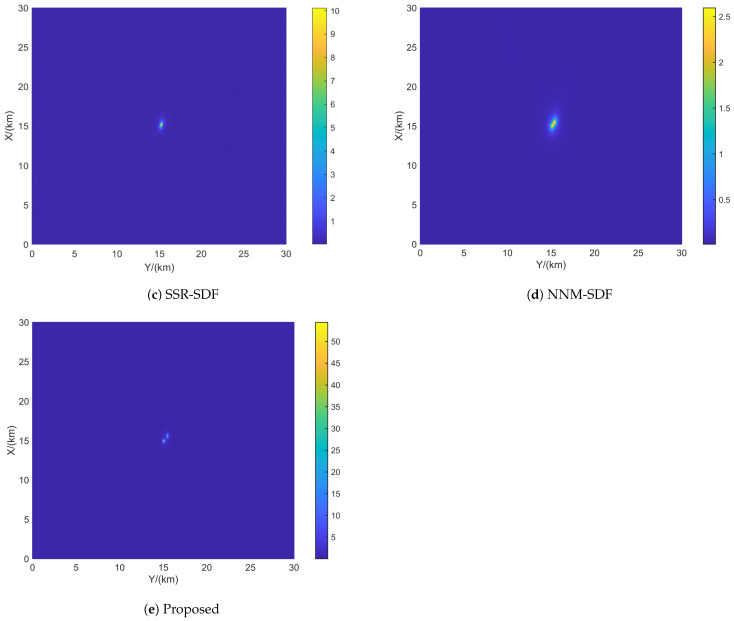
Resolution comparison in terms of the DPD spatial spectrum with the batch number L = 6 and SNR = 5 dB. (**a**) ULA-SDF algorithm. (**b**) SDCA-SDF algorithm. (**c**) SSR-SDF algorithm. (**d**) NNM-SDF algorithm. (**e**) Proposed algorithm.

From Figure 6, it can be seen that when SNR drops to 5 dB, only the proposed algorithm maintains good resolution. This is because the NNM algorithm does not account for the effects of noise, and the SSR algorithm, due to its grid-based approach, experiences energy aliasing between closely spaced grid points at lower SNR levels.

Table 1 shows the RMSEs of the two closed signal sources depicted in Figure 5 and Figure 6.

### 4.2. Localization Accuracy

This subsection shows the experiments’ root mean square error (RMSE) variation curves as a function of the SNR and snapshots. The RMSE is defined as in Equation (49):

(49) $$\mathrm{RMSE}=\sqrt{\frac{1}{N_{m}Q}\left(\sum_{q=1}^{Q}\sum_{i=1}^{N_{m}}{∥{\hat{p}}_{q}-p_{q}∥}_{2}^{2}\right)}$$

In Equation (49), $N_{m}$ represents the number of Monte Carlo trials in the experiment, and Q represents the number of actual signal sources. In this experiment, there are seven signal sources, and the number of Monte Carlo trials is set to 500.

**Figure 7:**
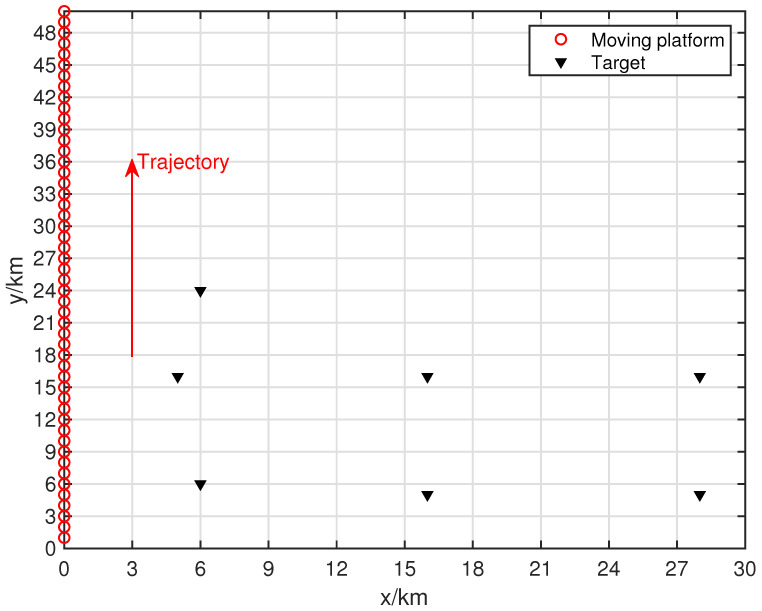
Moving platform scene related to seven targets.

Figure 7 shows the target and movement settings for the experiments in this section, where the number of movement batches is L=50. The search range for this algorithm was set to a rectangular area of (0 km, 30 km) × (0 km, 30 km), and a multi-level grid search strategy was used, with the finest grid resolution reaching 10 m × 10 m. The target positions in the scenario were set at (6, 6), (16, 5), (28, 5), (5, 16), (16, 16), (28, 16), and (6, 24) (all in km). The first two targets emit QPSK signals, the third target emits a UQPSK signal with a non-circularity rate of 0.8, and the remaining sources emitted BPSK signals.

**Figure 8:**
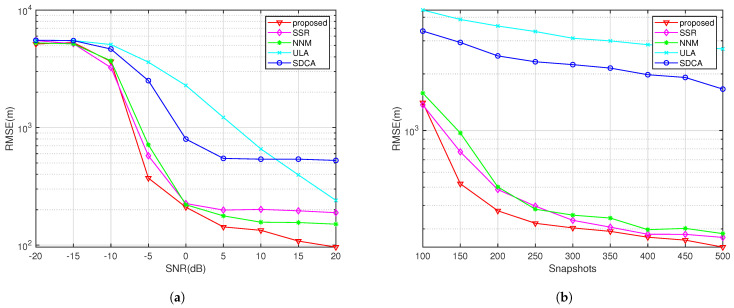
RMSE performance comparison with seven sources in SNR and snapshots. (**a**) K = 200. (**b**) SNR = −5.

From Figure 8a, it can be observed that the proposed algorithm exhibits excellent SNR performance, with increasingly precise localization as the SNR increases. Before approximately 12 dB, the uniform linear array (ULA) shows the worst localization performance, as it is constrained by the array aperture, preventing it from achieving better accuracy compared to sparse arrays. The SDCA-based localization algorithm consistently performs worse than the three sparse recovery algorithms, primarily because the SDCA algorithm discards more equivalent elements, resulting in less information being used compared to the sparse recovery algorithms. When the SNR exceeds −5dB, there remains a localization accuracy gap of several hundred meters compared to the algorithm proposed in this paper. At higher SNRs (after approximately 12 dB), this issue of lost information becomes more pronounced, with SDCA even performing worse than the ULA-based localization algorithm. Sparse recovery algorithms, which utilize all array element information, outperform the other algorithms.

It is also evident that once the SNR exceeds 5 dB, the curve for the SSR algorithm flattens, and the localization accuracy remains around 200 m. This occurs because the SSR algorithm uses a grid-based recovery strategy, and as the SNR becomes sufficiently high, the predefined grid increasingly fails to align with the true source locations, leading to the so-called “basis mismatch” problem, a major issue with grid-based algorithms. The localization performance of the NNM algorithm is inferior to that of the proposed algorithm by several hundred meters at lower signal-to-noise ratios. At higher SNRs, its localization accuracy is about 50 m worse than that of the proposed algorithm. This is because, although it uses a gridless recovery strategy, it does not account for the impact of noise, leading to greater errors in the recovered array elements compared to the other sparse recovery algorithms.

Figure 8b shows the RMSE curves of each algorithm as a function of the number of snapshots when the SNR is set to −5 dB. It can be observed that the ULA-based algorithms and the SDCA-based algorithm exhibit much worse localization accuracy compared to the sparse recovery algorithms. However, the situation where the ULA outperforms the SDCA, as seen in Figure 8a, does not occur here. In fact, the localization accuracy of the uniform linear array is approximately one kilometer lower than that of the SDCA-based algorithm. This indicates that, at low SNRs, the information discarded by the SDCA algorithm is less sensitive to changes in the number of snapshots.

At lower snapshot counts, the proposed algorithm has a significant advantage over the other algorithms, with its localization accuracy being approximately 100 m better than the other two sparse recovery algorithms. When the number of snapshots increases, the recovery performances of the three sparse recovery algorithms become similar. However, since the SSR algorithm involves predefined grid operations, its curve flattens after 400 snapshots.

### 4.3. The Impact of the Movement Trajectory

#### 4.3.1. Batch Number

**Figure 9:**
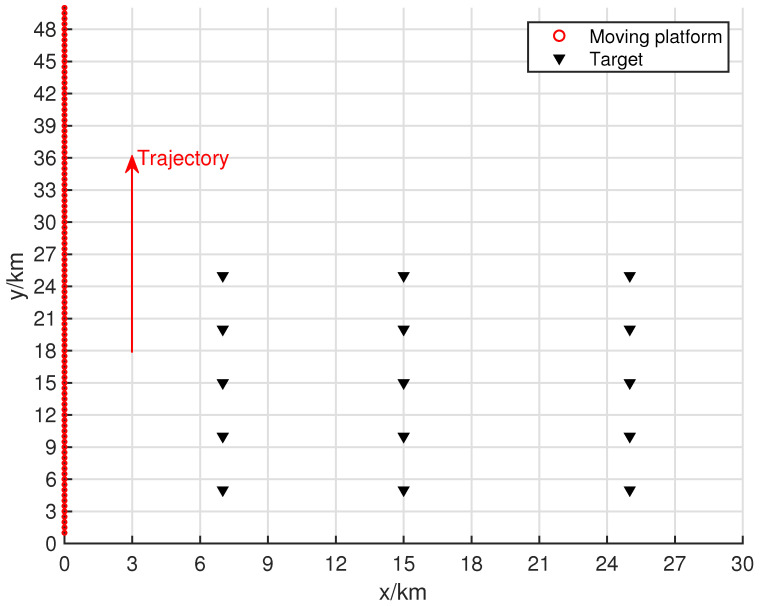
Moving platform scene related to 15 targets.

This section primarily investigates the impact of the movement trajectory and sampling batches on multi-target localization. Figure 9 shows a schematic of the simulation scenario. The true target positions are located at (7, 5), (7, 10), (7, 15), (7, 20), (7, 25), (15, 5), (15, 10), (15, 15), (15, 20), (15, 25), (25, 5), (25, 10), (25, 15), (25, 20), and (25, 25). Among these, the 6th, 7th, and 8th sources transmit QPSK signals, the 9th and 10th sources transmit UQPSK signals with a non-circularity rate of 0.8, and the remaining sources transmit BPSK signals. The SNR in the scenario is set to 20 dB, and the number of snapshots is K=200.

**Figure 10:**
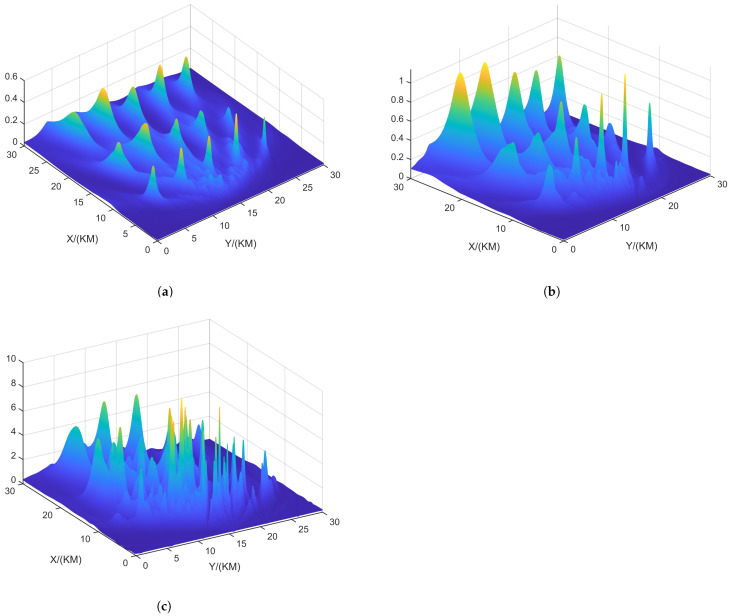
Localization performance with different batch number L, where SNR = 20 dB and K = 200. (**a**) Batch number = 99. (**b**) Batch number = 50. (**c**) Batch number = 10.

Figure 10 shows the localization performance for different batch numbers. It is evident that as the batch number decreases, the localization performance degrades significantly. When the batch number is reduced to 10, the sources become completely unrecognizable, with numerous false peaks appearing. This occurs because the reduction in batch number undermines the effectiveness of covariance fusion and the completeness of observations, leading to false peaks at the intersections of line-of-sight vectors (the DOA vectors generated at each observation point). As the number of sources to be estimated increases, the corresponding batch number should also increase.

#### 4.3.2. Movement Trajectory

This section will demonstrate the impact of different movement trajectories of the platform on localization performance. Several typical scenarios will be set up for a detailed explanation. In each scenario, the batch number *L* was set to 50, all sources emit BPSK signals, and the SNR and the number of snapshots for each source were provided, respectively.

**Figure 11:**
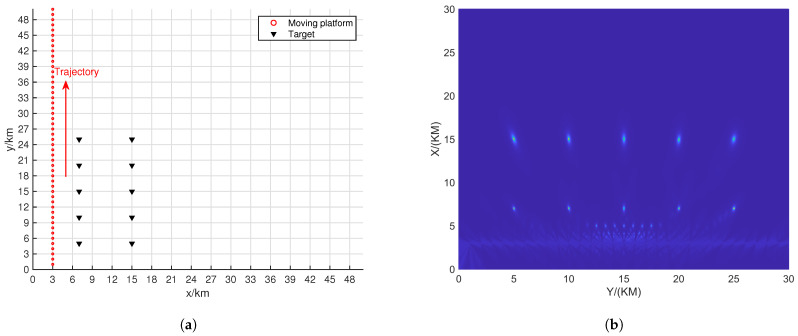
Motion scene along the y = 3 trajectory with SNR = 20dB and K = 200. (**a**) Motion scene. (**b**) Positioning results.

Figure 11a shows a scenario where the movement trajectory is close to the targets. From the localization results displayed in Figure 11b, it can be observed that many false peaks appear between the trajectory and the true target positions. A reasonable explanation is that when the trajectory is close to the targets, the intersection of different line-of-sight vectors increases, particularly resulting in more false peaks in the middle of the trajectory and the observation weights in the middle of the trajectory are higher, which leads to more false peaks in this region.

**Figure 12:**
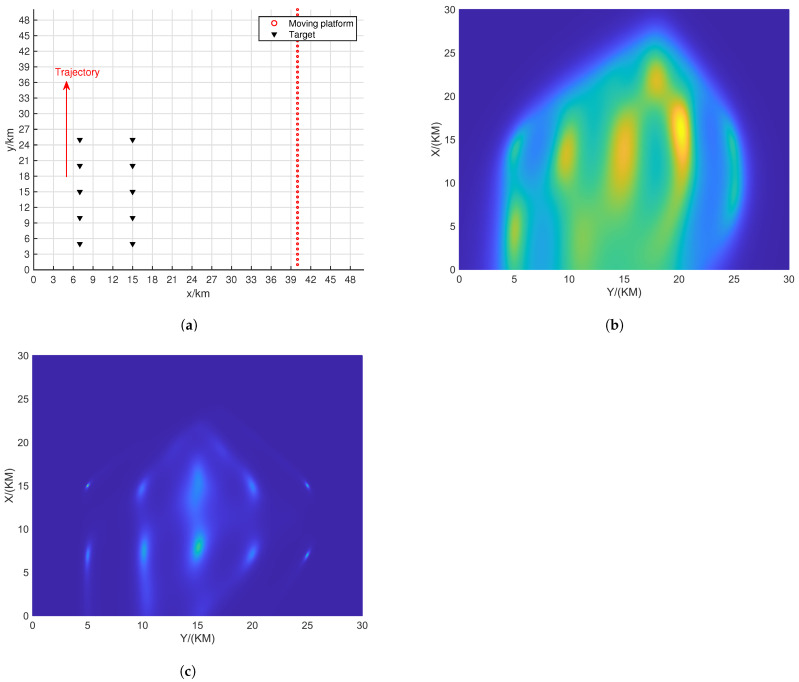
Motion scene along the y = 40 trajectory. (**a**) Motion scene. (**b**) Positioning results with SNR = 20 dB and K = 200. (**c**) Positioning results with SNR = 30 dB and K = 200,000.

Figure 12a shows a scenario where the movement trajectory is farther from the target scene. From the localization results displayed in Figure 12b, it can be seen that the resolution of the targets is extremely poor. This is mainly due to the resolution limitations of the MUSIC algorithm. At farther distances, the line-of-sight vectors become wider, causing different vectors to overlap at the target locations. Increasing the SNR and the number of snapshots, as shown in Figure 12c, results in better localization performance.

**Figure 13:**
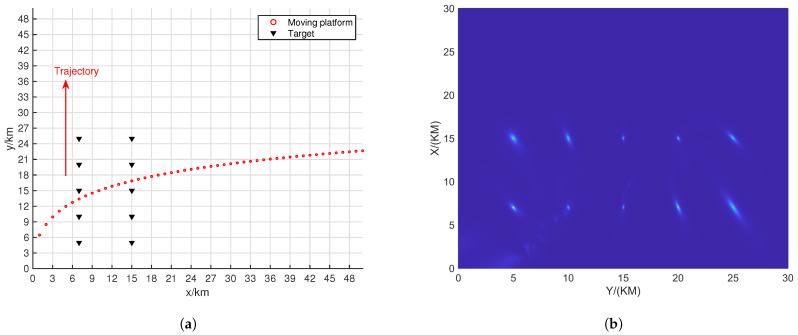
The curved trajectory passing through the target with SNR = 20 dB and K = 200. (**a**) Motion scene. (**b**) Positioning results.

Figure 13a shows a scenario where the trajectory is a curve passing through the targets. It can be observed that the localization performance varies for different targets. Targets farther from the trajectory exhibit wider spectral peaks, while targets closer to the trajectory have narrower spectral peaks. This indicates that the trajectory has a significant impact on the localization of the targets.

**Figure 14:**
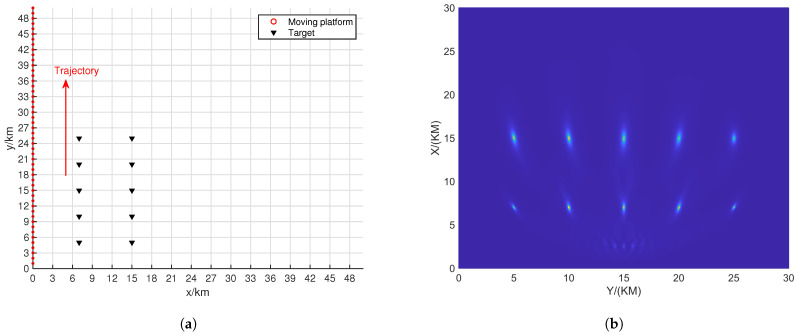
Motion scene along the y-axis trajectory with SNR = 20 dB and K = 200. (**a**) Motion scene. (**b**) Positioning results.

Figure 14a shows a scenario where the trajectory is along the y-axis. From Figure 14b, it can be seen that the localization performance for each point is good, and the amplitude of the false peaks is relatively low. This trajectory effectively handles the multi-target situation.

### 4.4. Degrees of Freedom

This section will demonstrate the array degrees of freedom for different algorithms. The scenario setup is the same as in Figure 9, with 99 movement batches, an SNR of 20 dB, and the number of snapshots set to K=200. The mixed signal model is also the same as in Figure 10. From Equation (39), we know that $\mathrm{rank}(R_{S_{wz}})=w+2z$, and according to the theory of the MUSIC algorithm, the maximum theoretical number of identifiable targets for the proposed algorithm is w+2z≤2∗J−1=25.

**Figure 15:**
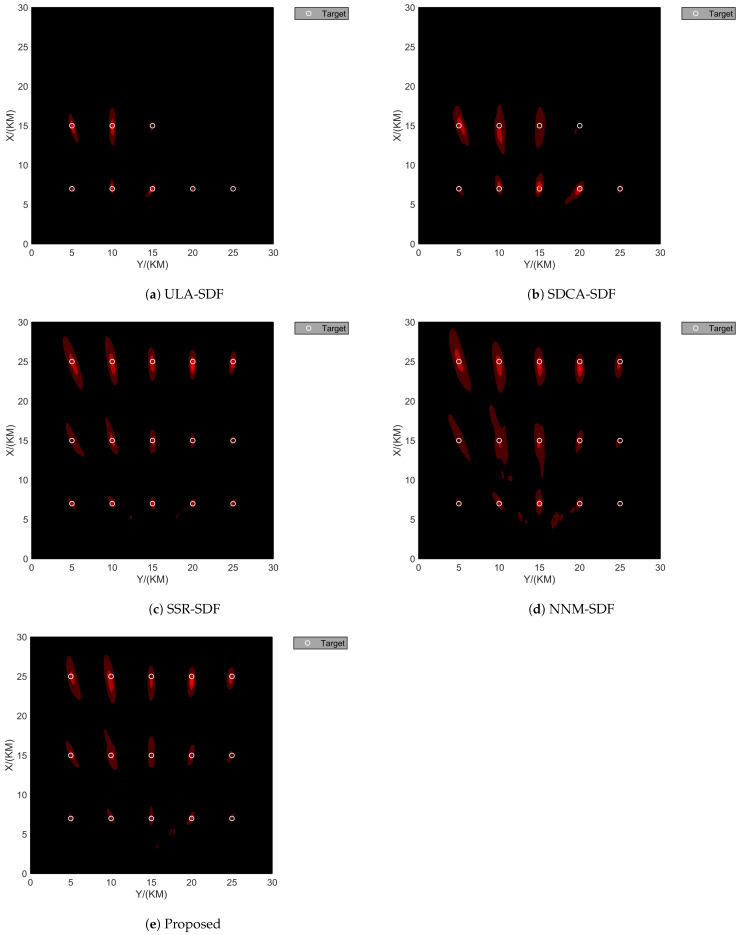
DOF comparison in terms of the DPD spatial spectrum with the batch number L = 99, SNR = 20 dB, and snapshots K = 200. (**a**) ULA-SDF algorithm. (**b**) SDCA-SDF algorithm. (**c**) SSR-SDF algorithm. (**d**) NNM-SDF algorithm. (**e**) Proposed algorithm.

Figure 15 shows the localization performances of different algorithms for multi-target scenarios. In Figure 15a, the algorithm is limited by the array aperture, and the maximum number of identifiable targets is w+2z≤13. Therefore, the last seven targets were removed, resulting in a used degree of freedom of w+2z=11. In Figure 15b, the algorithm only uses the largest continuous portion of the sum and difference coarrays, allowing it to identify a maximum of w+2z≤15 targets, so the last six targets were removed, with a used degree of freedom of w+2z=13.

For the sparse recovery algorithms shown in the figure, all algorithms fully utilize the array degrees of freedom of the sparse array. Among these, the proposed algorithm and the SSR algorithm demonstrate better localization performance than the NNM algorithm.

### 4.5. Computation Time

This section presents the final experiment in this paper, which simulates the computation time of each algorithm. The simulation was conducted on a system equipped with a 13th Gen Intel(R) Core(TM) i9-13900K CPU and 32.0 GB*2 RAM. The manufacturer of the CPU is Intel Corporation, located in Santa Clara, CA, USA. The simulation scenario is the same as that in Figure 6 from the first experiment. Each data point underwent 200 Monte Carlo trials, and all optimization problems involved in the simulation were solved using Matlab’s built-in CVX solver.

Table 2 shows the differences in computational complexity between the various algorithms. It can be observed that the sparse recovery algorithms have significantly higher complexity, mainly due to the optimization problem solving involved. The SSR algorithm has the highest complexity, primarily because of the grid-based solving process.

## 5. Discussion

### 5.1. The Influence of Target Circularity Balance

This section primarily considers the effect of significant imbalances between circular and non-circular signals emitted by multiple sources on the localization accuracy of the algorithm. The movement and localization scenario for this section is shown in Figure 7. In all simulation curves presented in this section, the number of snapshots is fixed at 200.

**Figure 16:**
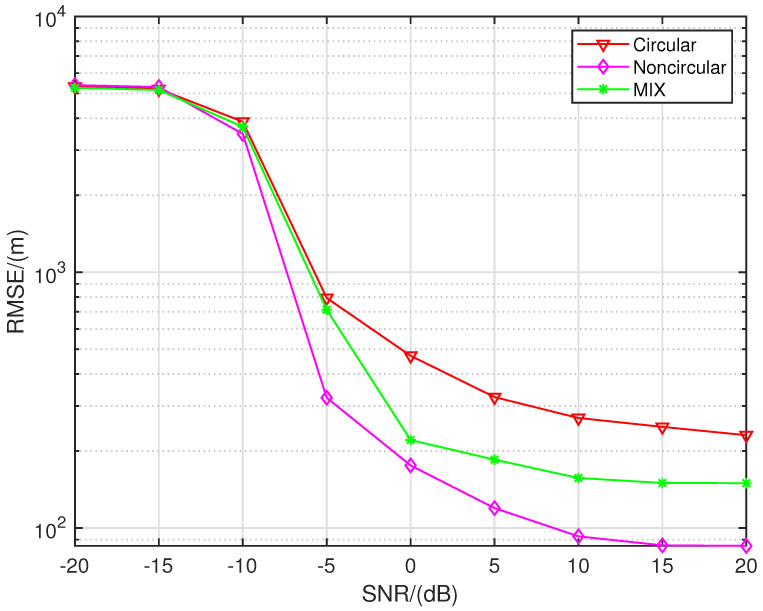
RMSE performance comparison with seven sources with different SNRs.

In Figure 16, the red curve represents the case where all seven signal sources emit circular signals, while the pink curve represents the case where all seven sources emit non-circular signals. The green curve depicts a scenario where the sources emit a mix of circular and non-circular signals: the target at (6, 6) emits a QPSK signal, while the sources at (16, 5) and (28, 5) (in kilometers) emit UQPSK signals with a non-circularity rate of 0.8, and the remaining sources emit BPSK signals. From Figure 16, it can be observed that as the proportion of non-circular signals increases, the localization accuracy improves. We will explain that this phenomenon is due to the fact that non-circular signals offer higher localization precision.

**Figure 17:**
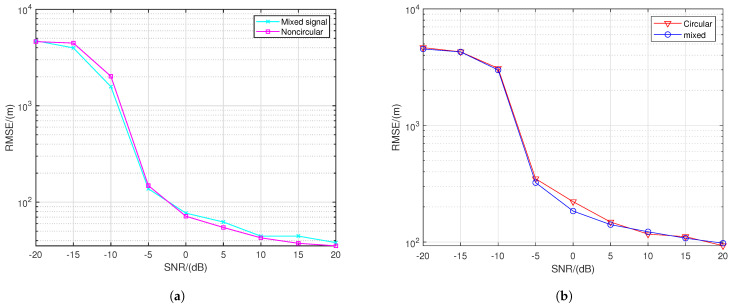
RMSE performance comparison with three sources with different SNRs. (**a**) Non-circular signal. (**b**) Circular aignal.

Figure 17 aims to show the RMSE curves of circular or non-circular signals under the influence of other signals in the detection area. We keep the circular or non-circular nature of the signal sources at three locations—(6, 6), (16, 5), and (5, 16) (in kilometers)—unchanged while varying the circular or non-circular nature of the signals from the other positions. In Figure 17a, the three designated targets emit BPSK signals, with the indigo curve representing the scenario where the remaining four targets emit QPSK signals, and the pink curve representing the scenario where the remaining four targets emit BPSK signals. It can be observed that the localization accuracy of non-circular signals is almost unaffected by the circular or non-circular nature of the signals from other positions.

Similarly, in Figure 17b, the three designated targets emit QPSK signals, with the blue curve representing the case where the remaining four targets emit BPSK signals, and the red curve representing the case where all targets emit QPSK signals. It can be seen that the localization accuracy of circular signals is also unaffected by the circular or non-circular nature of the signals from other positions. Moreover, the RMSE of localization in Figure 17a is lower than that in Figure 17b, indicating that non-circular signals provide higher localization accuracy than circular signals. This is because the unconjugated covariance matrix of non-circular signals is non-zero, allowing the use of both the difference coarray and sum coarray information. In contrast, the unconjugated covariance matrix of circular signals is zero, meaning less information is available compared to non-circular signals.

### 5.2. The Measurement of Low SNR

This section considers the localization performance of the algorithm in low-signal-to-noise-ratio (SNR) conditions, as well as the measures taken to ensure the algorithm’s effectiveness in such scenarios. The movement and localization scenario, along with the type of signals emitted by each source, are the same as those shown in Figure 7.

**Figure 18:**
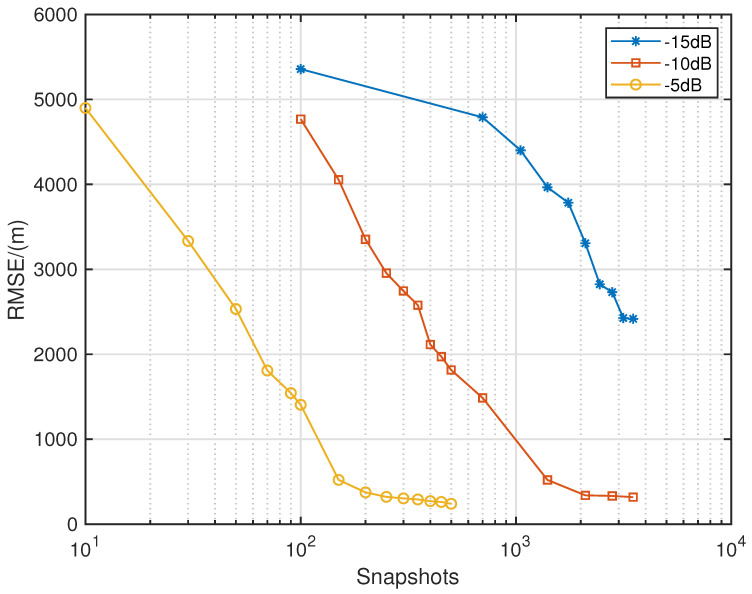
RMSE performance comparison with seven sources in different snapshots.

Figure 18 shows the RMSE variation curves as a function of the number of snapshots under low-SNR conditions. The SNR information for each curve is indicated in the figure. As shown in Figure 18, when the SNR decreases, the curve shifts to the right on the graph with the number of snapshots on the horizontal axis. The curves for −10 dB and −5 dB exhibit an almost parallel shift, while the blue curve for −15 dB shows a similar shift trend in the overlapping regions with the other two curves. This indicates that the localization accuracy of the proposed algorithm decreases as the SNR decreases. However, by increasing the number of snapshots, a good localization performance can still be achieved.

## 6. Conclusions

This paper establishes a model for motion-based single-station direct localization using narrowband signals, integrating a sparse array on the platform to achieve higher localization accuracy. To enhance the array’s degrees of freedom, the unconjugated covariance matrix of non-circular signals, which is non-zero, is leveraged to expand the array’s degrees of freedom, introducing the SDCA. The array interpolation method is then used to fill in the gaps in the SDCA, forming a virtual full array. Through analyzing the covariance of the virtual full array and the differences in non-circularity rates of non-circular signals, the direct localization spectral function is successfully derived. This paper also applies a unitary transformation to shift the spectral function search process from the complex domain to the real domain, significantly reducing computation time. Finally, detailed simulation experiments were conducted to validate the algorithm’s superiority in terms of localization accuracy, resolution, the impact of movement trajectories, and computational complexity. In the scenario set in this paper, the localization accuracy of the proposed algorithm surpasses other algorithms by several hundred meters in low-SNR conditions. In high-SNR conditions, it is tens of meters better than other sparse recovery algorithms and outperforms traditional algorithms by several hundred meters. Considering the advantages of single moving stations in terms of equipment cost and localization accuracy, this system has broad application prospects in civilian radiation source localization, such as in navigation interference and communication jamming. Future work will include the practical validation of the proposed algorithm in these applications.

## Figures and Tables

**Table 1:** Resolution RMSE comparison with different algorithms (unit per kilometer).

Algorithm	SNR = 5 dB	SNR = 10 dB
ULA-SDF	0.3210	0.2973
SDCA-SDF	0.2937	0.2820
SSR-SDF	0.2933	0.2332
NNM-SDF	0.2884	0.2217
Proposed	0.2742	0.2062

**Table 2:** Computation time comparison with different algorithms.

Algorithm	Algorithm Time	Exhaustive Search Time
ULA-SDF	0.013367	7.016410
SDCA-SDF	0.018876	7.201545
SSR-SDF	6.443729	9.073410
NNM-SDF	4.048454	9.062430
Proposed	4.470362	9.050351

## Data Availability

Data are contained within the article.
